# Socioeconomic Inequalities in Home-Care Use Across Regional Long-term Care Systems in Europe

**DOI:** 10.1093/geronb/gbaa139

**Published:** 2020-09-30

**Authors:** Ginevra Floridi, Ludovico Carrino, Karen Glaser

**Affiliations:** Department of Global Health & Social Medicine, King’s College London

**Keywords:** Long-term care, Multilevel models, SHARE, Socioeconomic status

## Abstract

**Objectives:**

We examine whether socioeconomic inequalities in home-care use among disabled older adults are related to the contextual characteristics of long-term care (LTC) systems. Specifically, we investigate how wealth and income gradients in the use of informal, formal, and mixed home-care vary according to the degree to which LTC systems offer alternatives to families as the main providers of care (“de-familization”).

**Method:**

We use survey data from SHARE on disabled older adults from 136 administrative regions in 12 European countries and link them to a regional indicator of de-familization in LTC, measured by the number of available LTC beds in care homes. We use multinomial multilevel models, with and without country fixed-effects, to study home-care use as a function of individual-level and regional-level LTC characteristics. We interact financial wealth and income with the number of LTC beds to assess whether socioeconomic gradients in home-care use differ across regions according to the degree of de-familization in LTC.

**Results:**

We find robust evidence that socioeconomic status inequalities in the use of mixed-care are lower in more de-familized LTC systems. Poorer people are more likely than the wealthier to combine informal and formal home-care use in regions with more LTC beds. SES inequalities in the exclusive use of informal or formal care do not differ by the level of de-familization.

**Discussion:**

The results suggest that de-familization in LTC favors the combination of formal and informal home-care among the more socioeconomically disadvantaged, potentially mitigating health inequalities in later life.

Governments in ageing societies are grappling with the issue of long-term care (LTC). Projected increases in disability mean that many countries face challenges in meeting the growing care needs of an ageing population (Colombo et al., 2011). LTC refers to policies that support older people with limitations in everyday activities (Colombo et al., 2011). In Europe, recent LTC reforms have aimed to curb expenditures by promoting a shift from LTC provided in care homes (i.e., care provided in non-acute residential and nursing facilities) to formal home-based care, increasing emphasis on family support (European Commission, 2018; Fernandez et al., 2016). Greater reliance on families through reductions in publicly provided or subsidized LTC services may restrict access to formal care especially among individuals from lower socioeconomic status (SES) groups. This is because poorer individuals are less able to purchase formal care on the market than the rich, and consequently more likely to rely exclusively on informal care, typically provided by families (Agree and Glaser, 2009; Carrino et al., 2018; Suanet et al., 2012). This may act to exacerbate socioeconomic inequalities in care use and provision across families. To date, it remains unclear whether and how LTC system characteristics are associated with socioeconomic inequalities in informal and formal home-care use.

European LTC systems differ with respect to de-familization, defined as the degree to which the state or the market, as opposed to families, take responsibility for the provision of care (Leitner, 2003; Saraceno, 2016; Saraceno and Keck, 2010). While socioeconomic gradients in (in)formal (i.e., formal and informal) home-care use appear to vary across countries according to the level of de-familization in LTC, evidence from comparative studies is scant, and limited to comparing groups or typologies of countries (Albertini and Pavolini, 2017; Broese van Groenou et al., 2006; Carrieri et al., 2017). Our research addresses two major gaps in the literature on care inequalities. First, by testing for interactions between individual SES indicators and LTC system characteristics, we examine whether de-familization is associated with inequalities in (in)formal home-care use among community-dwelling disabled older adults. Second, we exploit within-country regional variation in LTC systems. This represents a considerable improvement over country-level comparisons because, in many European countries, LTC characteristics vary substantially across administrative regions (Eurostat, 2019). This study advances previous research linking inequalities in care to features of LTC systems (Albertini and Pavolini, 2017; Broese van Groenou et al., 2006). It is timely as, throughout Europe, LTC systems rely increasingly less on formal care provision, either in care homes or through formal home-care (European Commission, 2018).

We conceptualize informal care as personal care from kin and non-kin, and formal home-care as paid home-care from public or private providers. Given the interdependent nature of these forms of care (Bonsang, 2009; Motel-Klingebiel et al., 2005), we analyze them simultaneously to provide a comprehensive understanding of inequalities in home-care use. Using 2015 data from the Survey of Health, Ageing and Retirement in Europe (SHARE), we investigate whether wealth and income gradients in (in)formal home-care use among older disabled Europeans relate to the number of available LTC beds in care homes across 136 regions in 12 countries. We consider high numbers of LTC beds as indicative of de-familization, in contrast to familism, which refers to LTC settings where the family is assumed to be the main provider of care (Saraceno, 2016).

## Individual-Level Inequalities in Care Use

Andersen and Newman’s (2005) behavioral model of health care suggests that socioeconomic differences in care use reflect differences in individual needs, predisposing and enabling factors (Broese van Groenou et al., 2006). Physical and cognitive health determine needs for care, which are generally higher among individuals in lower-SES groups (Ilinca et al., 2017). Gender and age represent predisposing factors for care, and their distribution varies by SES (Broese van Groenou et al., 2006). Enabling factors refer to individuals’ social, material, and financial resources. These include education, which may enhance individuals’ ability to navigate the care system; family structure, which determines the availability of potential caregivers (Broese van Groenou et al., 2006); and ownership of material resources such as a home or car, which can facilitate care provision or access (Vlachantoni et al., 2015). Last, financial resources relate to care use primarily by influencing individuals’ ability to purchase care (Rodrigues et al., 2018). The model predicts that once health, predisposing factors, social and material resources are controlled for, any residual socioeconomic disparities in care use solely reflect differences in financial resources (Rodrigues et al., 2018).

Wealth and income capture financial resources. Financial wealth is generally a better indicator of SES for those over age 60 (Robert and House, 1996; Tanaka et al., 2012). This is because income tends to reduce considerably after retirement, while wealth captures the cumulative effects of lifetime advantages and disadvantages with respect to material resources (Kaplan et al., 1987; Robert and House, 1996). We use both financial wealth and income as indicators of financial resources. Financial wealth may be strongly associated with informal care use through its connection to family resources and intergenerational transfers, which may be made in exchange for care (Rodrigues et al., 2018). Being more readily disposable than wealth, income may have a stronger impact on the ability to purchase formal home-care (Rodrigues et al., 2018).

Empirical evidence on the association between SES and care use is inconclusive. While some research finds that, across Europe, those in lower-SES groups are more likely to receive informal (Broese van Groenou et al., 2006; Vlachantoni et al., 2015) or formal support (Rodríguez, 2014), other studies find that those in higher-SES groups report higher use of both informal (Bakx et al., 2015; Rodríguez, 2014) and formal care (Albertini and Pavolini, 2017). The conflicting evidence has been partly explained by differences in the socioeconomic indicators used, such as income versus wealth (Rodrigues et al., 2018). Other scholars argue that socioeconomic inequalities in care use play out differently across different LTC systems (Albertini and Pavolini, 2017). However, as we argue below, evidence from comparative research is limited.

## De-familization, Familism, and Inequalities in Care Use

LTC encompasses both informal care (from family or non-kin) and formal care (formal home-care or in care homes). European LTC systems differ in the extent to which the responsibility for care lies with the state, the market, or the family (Kalmijn and Saraceno, 2008). LTC systems characterized by de-familization (equivalently, “de-familized” LTC systems) offer alternatives to informal care, thereby reducing family responsibility. By contrast, familism refers to settings where policies, cultural norms, and preferences emphasize the family as the sole or main provider of care (equivalently, “familistic” contexts) (Kalmijn and Saraceno, 2008; Leitner, 2003; Saraceno and Keck, 2010). In familistic contexts, family support often involves intensive care, including daily personal care (Brandt et al., 2009). Norms and obligations around family care also tend to be stronger than in de-familized contexts (Leitner, 2003; Saraceno and Keck, 2010), where family support is more frequently directed towards help rather than personal care (Brandt et al., 2009). While previous studies have broadly classified Northern European countries as de-familized and Southern European countries as familistic (Brandt et al., 2009), the wide fragmentation of LTC policies even within countries suggests that country-level LTC typologies risk being overly simplistic (European Commission, 2018; Saraceno and Keck, 2010). We add to the literature by considering features of the LTC systems at the subnational (regional) level.

The degree of de-familization in LTC may shape the distribution of (in)formal home-care use by SES by determining the extent to which individuals of different SES are able to access formal care (Saraceno, 2010). Higher de-familization may alleviate socioeconomic inequalities in formal or mixed home-care use by providing alternatives to family care among lower-SES groups. In contrast, lower de-familization (i.e., higher familism) may lead to exclusive reliance on informal care especially among poorer individuals, who may be unable to afford alternatives to family care (Saraceno, 2010). Interactions between LTC system characteristics and individual SES have not been previously tested.

## Empirical Evidence

Few studies analyze inequalities in (in)formal home-care from a comparative perspective, showing mixed results. Motel-Klingebiel et al. (2005) find no socioeconomic gradient in informal care use in any of the countries studied, which have varying levels of de-familization (England, Germany, Israel, Norway, and Spain). Broese van Groenou et al. (2006) find that the poor are more likely than the rich to use informal care from outside the household in Britain and the Netherlands, but not in Italy and Belgium (relatively less de-familized countries). Studies on inequalities in formal home-care use find that those with higher incomes are more likely to use formal home-care than poorer individuals in countries where de-familization is low (Italy and Germany), but not where de-familization is high (Denmark and the Netherlands) (Albertini and Pavolini, 2017; Bakx et al., 2015). Ilinca et al. (2017) find that the poor are relatively more likely to use formal care than the rich in Denmark and the Netherlands, but not elsewhere in Europe. Carrieri et al. (2017) compare formal care inequalities across three European regions and find that the rich use more formal care than the poor in Southern and Continental Europe, but not in Northern Europe, where LTC expenditure is higher (indicating higher de-familization).

All the studies noted above either compare a few countries, providing qualitative descriptions of their LTC systems (Albertini and Pavolini, 2017; Broese van Groenou et al., 2006), or cluster countries into macro-groups (e.g., North and South), concealing considerable within-group variation (Carrieri et al., 2017). Therefore, these studies are unable to explicitly estimate interactions between LTC system characteristics and individual SES in determining (in)formal home-care use. Moreover, the approach of taking countries as the primary units of comparison disregards substantial internal variation and fragmentation in LTC systems. For example, service availability and eligibility rules for public LTC vary greatly across administrative regions in countries like Belgium or Italy (Brugiavini et al., 2017; Eurostat, 2019).

## Aims and Hypotheses

We analyze wealth and income gradients in informal and formal home-care use by disabled individuals aged 65+, and we compare these gradients across regions with different degrees of de-familization in LTC. Following previous literature on care inequalities (Rodrigues et al., 2018), we refer to SES gradients as being either “pro-poor” (indicating that the poor receive more of a certain type of care than the rich) or “pro-rich” (indicating that the rich receive more of a certain type of care than the poor). We are ultimately interested in whether wealth and income gradients in informal, formal and mixed-care become *relatively* more pro-poor or *relatively* more pro-rich as the level of de-familization in LTC varies across regions. Our use of these terms carries no normative connotation: for example, if the SES gradient in the exclusive use of informal care is “more pro-poor” in a certain region (relative to another), it is not necessarily beneficial to the well-being of the poor in that region, as it may indicate that they are less able to access formal home-care. Our three hypotheses about how socioeconomic inequalities in informal and formal home-care vary across areas are:

- H-a: *The higher is the level of de-familization, the less pro-poor is the SES gradient in the exclusive use of informal care*. Greater availability of formal care may make the poor relatively less likely to rely only on informal care compared to the rich.
- H-b: *The higher is the level of de-familization, the more pro-poor is the SES gradient in the exclusive use of formal home-care*. Greater availability of formal care may facilitate access to formal home-care among the poor more than among the rich (e.g., by reducing the cost of formal home-care).
- H-c: *The higher is the level of de-familization, the more pro-poor is the SES gradient in the exclusive use of mixed (i.e., both formal and informal) care*. Higher de-familization may facilitate access to formal home-care for poorer (compared to richer) individuals, without decreasing their reliance on informal care networks.

Drawing on earlier work showing how wealth and income relate to care use (Rodrigues et al., 2018), we also hypothesize that:

- H-d: *On average, wealth has a stronger association with the exclusive use of informal care than income*, as it may affect such care through intergenerational transfers and bequests.
- H-e: *On average, income has a stronger association with the exclusive use of formal care than wealth*, as it may more directly affect one’s ability to purchase such care.

We employ information on the number of beds available in care homes by region (“LTC beds” henceforth; Wagner and Brandt, 2018). In particular, higher LTC beds availability indicates higher de-familization, and lower LTC beds availability indicates lower de-familization. De-familization in LTC systems may occur through two channels: LTC provision in care homes (public or privately paid) and formal home-care provision (public or privately paid). Our LTC beds indicator captures the first of these components. Ideally, it would be complemented by an indicator of formal home-care provision, such as expenditure on home-care services. However, comparative data on home-care services are not available at the regional level in Europe.

It should be emphasized that our analysis relates to (in)formal care received at home in a community-dwelling sample (excluding individuals living in care homes). A potential concern is that LTC beds are inversely related to formal home-care, as regions with fewer LTC beds may invest more resources in formal home-care services. While comparative regional-level data on home-care services are not available to directly test this, in Supplementary Table 1a and b, we show that LTC beds are strongly and positively correlated with public expenditures on both care homes (+0.83) and formal home-based care (+0.62) across the countries in our sample. If the same holds true at regional level, LTC beds offer several advantages as an indicator of de-familization.

First, among different forms of LTC, care in care homes is highly “external” to the family, as it is provided full-time and is typically less reliant on family involvement (Saraceno and Keck, 2010). As such, it represents a stronger form of de-familization compared to formal home-care services, which are usually mediated by family members (e.g., organizing and co-providing care). Second, LTC beds indicate availability of LTC services as opposed to measures of service use (e.g., percentage of population receiving care), which would more strongly correlate with the same unobserved characteristics related to (in)formal care use, our outcome of interest. Third, LTC beds are relatively stable over time (Eurostat, 2019), suggesting it is a good proxy for the structural characteristics of LTC systems as opposed to year-specific population structure or macro-economic factors such as gross domestic product (GDP) (which we control for in the analysis). Finally, LTC beds are, to the best of our knowledge, the only comparative LTC indicator available at regional level (Eurostat, 2019), allowing us to exploit variation in LTC systems across and within countries.

## Method

### Data and Sample Selection

We analyze data from the sixth wave (2015) of SHARE, a multidisciplinary survey representative of individuals aged 50 and older not living in care homes across Europe (Börsch-Supan et al., 2013; Börsch-Supan and Jürges, 2005). It contains information on respondents’ demographic, socioeconomic, and health characteristics, and on their use of home-care from informal and formal providers. Since our outcome of interest is care use, we restrict our analytic sample to disabled individuals (Rodríguez, 2014), defined as respondents aged 65+ who report long-term difficulties performing at least one of a set of 23 activities including activities of daily living (ADL), instrumental ADL (IADL) and mobility items. A list of these activities is presented in Supplementary Appendix 1. Our results are robust to using alternative definitions of disability (Supplementary Tables 7–12). Since SHARE is not representative of the population living in care homes, we focus on home-care use only. We exclude 157 respondents who live in a care home and/or report receiving temporary nursing-home care in the past year. In addition, we only use observations from 12 countries for which regional-level LTC indicators are available from Eurostat (2019); namely, Austria, Belgium, Croatia, Czech Republic, Estonia,^1^ France, Germany, Italy, Poland, Spain, Sweden, and Switzerland. Our analytic sample consists of 15,403 individuals.

### Measures

#### Outcome

We define care use as the self-reported use of personal home-care in the previous 12 months. Personal care includes help with tasks such as dressing, walking, and eating, but excludes help with household chores or paperwork. Respondents give information on who provides care and the frequency of care use, from which we create a categorical variable indicating whether respondents report receiving: (a) *no care*; (b) exclusively *informal care* from any kin or non-kin (e.g., partners, children, friends) at least once per week^2^; (c) exclusively *formal care* in the form of professional or paid home-care; or (d) a combination of informal and formal care—*mixed-care*.

#### Income and wealth

Our main explanatory variables are financial wealth and income. Financial wealth is measured at household level as the sum of financial assets minus debts. This excludes housing wealth, defined as the value of all residential dwellings owned by the household, minus any debt owed on those dwellings. Income is measured at benefit-unit level (i.e., at couple level if with a partner). Both wealth and income are equivalised for household or benefit-unit composition (Hagenaars et al., 1994), and measured in Purchasing Power Parity (PPP) Euros for comparability across countries. We trim extreme values (the top and bottom 1%) and, following previous studies, apply the inverse hyperbolic sine transformation (arcsinh) to approximate a normal distribution (Bellemare and Wichman, 2020; Belloni et al., 2019). The arcsinh approximates the natural logarithm and allows retaining zero- and negative-valued observations (e.g., to capture debt). Coefficients on arcsinh-transformed variables can be interpreted similarly as coefficients on log-transformed variables, as we discuss in greater detail in Supplementary Appendix 2.

#### Individual-level control variables

Following our theoretical framework (Andersen and Newman, 2005) and previous studies (Albertini and Pavolini, 2017; Vlachantoni et al., 2015), in all the multivariate models, we control for predisposing factors (gender and age); need factors (ADL, IADL, and mobility limitations; diagnosed chronic conditions; poor self-rated health; and low cognitive function); social resources (education, marital status, and parental and child coresidence status); and material resources (home ownership and access to a car). The coding of these variables is outlined in Table 1.

**Table 1.** Descriptive Sample Characteristics, Overall and by Care Type

		By type of care received			
	Total	No care	Informal care only	Formal care only	Mixed-care
Predisposing factors					
Sex: female (%)	62.5	62.5	59.1	67.3	64.5
Age, years (mean)	75.7	74.8	78.1	81.1	81.3
Need factors					
ADL limitations ^a^ (%)					
None	73.6	84.8	27.8	41.4	13.5
1 limitation	13.2	10.4	26.9	24.5	19.0
2+ limitations	13.2	4.8	45.3	34.1	67.5
IADL limitations ^a^ (%)					
None	63.3	74.1	19.8	25.5	8.6
1 limitation	17.0	16.7	22.0	18.6	10.4
2+ limitations	19.7	9.2	58.2	55.9	81.0
No. of mobility limitations ^b^ (mean)	3.50	2.94	5.84	4.99	6.68
Any chronic conditions ^c^: yes (%)	82.2	80.0	91.0	87.3	92.6
Poor self-rated health: yes (%)	21.9	15.5	51.3	33.1	55.4
Low cognitive function ^d^: yes (%)	7.6	5.7	14.9	14.7	16.0
Social resources					
Education ^e^ (%)					
Lower secondary	52.6	50.2	63.2	59.9	62.5
Upper secondary	31.9	33.1	27.1	27.6	26.6
Tertiary	15.5	16.7	9.7	12.6	10.9
Marital status (%)					
Married	60.8	62.2	64.6	33.1	54.7
Never married	4.3	4.5	2.7	5.0	4.9
Separated or divorced	7.8	8.3	4.6	9.8	4.5
Widowed	27.0	25.0	28.1	52.1	35.9
Parental status ^f^ (%)					
Childless	9.6	9.2	7.6	16.6	13.3
Children outside household	73.1	74.0	66.8	76.2	67.6
Coresident child(ren)	17.4	16.7	25.6	7.2	19.1
Material resources					
Home ownership ^g^: yes (%)	70.7	71.8	70.8	58.5	62.2
Access to a car ^h^: yes (%)	57.2	61.8	43.1	29.5	35.2
Financial resources					
Financial wealth (PPP-adj.) (mean)	162,213	181,124	85,043	103,944	63,326
Income (PPP-adj.) (mean)	47,301	50,053	32,446	48,131	32,258
Sample sizes	15,403	12,395	1,635	683	691
Sample proportions		80.5	10.6	4.4	4.5

*Notes*: ADL = activities of daily living; IADL = instrumental activities of daily living; PPP = Purchasing Power Parity.

^a^Coded as whether respondent has 0 limitations; 1 limitation; or 2+ limitations (see Supplementary Appendix 1 for a list of activities).

^b^Min = 0 and max = 10 (see Supplementary Appendix 1 for a list of activities).

^c^One, if respondent reports any diagnosed chronic condition, excluding hypertension.

^d^One, if respondent has either a low memory score (i.e., <8 out of 20 words recalled) or a low time orientation score (i.e., two or more mistakes in identifying day of the week, date, month, and year).

^e^Coded using ISCED 1997: up to lower secondary (ISCED 0–2); upper secondary (ISCED 3–4); tertiary (ISCED 5–6).

^f^Coded as whether respondent is childless, has all children living outside household, or has at least one coresident child.

^g^One if the household owns the home where the respondent lives.

^h^One if anyone in the household where the respondent lives owns a car.

#### Regional-level indicators

Our macro-level indicator of de-familization is the number of LTC beds per 1,000 inhabitants in 2015, at regional level using the Nomenclature of Territorial Units for Statistics (NUTS-2) classification from Eurostat (2019).^3^ We also employ regional indicators for PPP-adjusted GDP per capita (in 1,000 Euros), and for the percentage of population aged 65+ in 2015 (Eurostat, 2019).

### Statistical Analysis

We conceptualize home-care use as the result of need, predisposing and enabling factors at the individual level (Andersen and Newman, 2005), and of LTC system de-familization at the regional level. We adopt a multinomial multilevel framework with individuals nested within regions, and regions nested within countries.

First, we fit a “baseline” random-effects model (M1b) of care use on all individual-level covariates and the macro-level indicator for LTC beds, as well as region and country random intercepts. These allow for the average level of each type of care to vary randomly across regions within countries, and across countries. In order to estimate how the probability of receiving each type of care varies by SES at different levels of de-familization in LTC, we add a cross-level interaction between wealth and LTC beds to the model (M1w), and do the same for income in a separate model (M1i). All models include individual-level characteristics as controls, coded as described in Table 1.

The multilevel random-effects model assumes that LTC beds are uncorrelated with the region and country random intercepts (Snijders and Bosker, 2012). However, this indicator is likely correlated with regional or country characteristics that also affect home-care use, such as population structure, spending on public services, and cultural norms around family caregiving (Saraceno and Keck, 2010). Consequently, the coefficient for the cross-level interaction may confound effects of care inequalities that are attributable to either LTC de-familization or to other unobserved macro-level factors. We therefore estimate more restrictive models that account for country-level unobserved heterogeneity by replacing the country random intercept with country fixed-effects. Moreover, since the number of beds is likely correlated with regional population structure and macro-economic characteristics, we control for regional GDP per capita and percentage of population aged 65+. We label these models as M2b (no interaction terms), M2w (interaction for wealth inequality), and M2i (interaction for income-inequality). These models exclude Switzerland, for which regional GDP data are unavailable. We perform all analyses using Stata 15.

## Results

### Descriptive Sample Characteristics

Table 1 shows descriptive sample statistics by type of care received. In line with previous research (Broese van Groenou et al., 2006; Vlachantoni et al., 2015), individuals receiving only informal care (10.6% of the sample) are more likely to be married, have coresident children and own their home, compared with formal or mixed-care users. Individuals receiving only formal care (4.4%) are disproportionately female and older, report less severe disability and better self-rated health. They have higher average financial wealth and income but are less likely to have a car in the household compared to other care recipients. Mixed-care users (4.5%) have worse health and lower financial wealth than the other groups, and lower income compared to those receiving only formal care.

In Table 2, we report descriptive statistics (by country) for our regional-level indicators of LTC beds, GDP per capita and the share of 65+ population. These statistics are obtained from Eurostat (2019) for the sample of 136 regions in 12 countries for which SHARE data are available. The average number of LTC beds across regions is 8.48/1,000 inhabitants, with large variation both across and within countries, for example, in Spain, Italy and Austria.

**Table 2.** Regional Sample Characteristics, Summary Statistics Calculated by Country

	Number of regions in the sample	LTC beds/1,000 inhabitants		GDP per capita (1,000s, PPP-adj.)		Percentage population 65+	
Country		Mean (*SD*)	Min–max	Mean (*SD*)	Min–max	Mean (*SD*)	Min–max
Austria	9	8.06 (2.18)	5.21–11.67	36.9 (6.73)	26.1–45.3	18.6 (1.56)	16.7–20.7
Belgium	11	11.89 (1.95)	7.75–15.10	32.9 (11.1)	21.9–59.3	17.8 (2.16)	13.1–22.1
Croatia	2	2.23 (0.09)	2.17–2.29	17.1 (0.71)	16.6–17.6	19.1 (1.24)	18.2–20.0
Czech Republic	8	7.03 (1.46)	3.84–8.53	25.4 (11.4)	18.8–53.3	17.8 (0.61)	16.7–18.3
Estonia	1	8.61 (n/a)	8.61–8.61	22.0 (n/a)	22.0–22.0	18.8 (n/a)	18.8–18.8
France	22	10.76 (2.75)	5.53–14.92	26.7 (5.89)	22.8–51.5	19.9 (2.54)	14.0–24.6
Germany	16	12.00 (1.69)	9.96–14.65	34.7 (9.51)	24.3–60.1	21.7 (1.92)	18.9–25.0
Italy	18	4.09 (2.81)	0.50–9.10	27.3 (7.38)	16.7–42.3	22.2 (2.51)	17.6–28.0
Poland	16	1.91 (0.27)	1.52–2.55	17.1 (2.76)	13.7–22.2	15.4 (1.03)	13.6–17.2
Spain	18	8.87 (4.79)	1.68–22.27	25.6 (5.32)	18.2–36.1	18.8 (3.50)	11.1–24.0
Sweden	8	13.80 (2.38)	10.04–17.02	34.0 (7.27)	28.9–51.4	20.7 (2.50)	15.7–23.4
Switzerland	7	11.75 (1.56)	8.62–13.42	Not available	Not available	18.1 (1.71)	16.7–21.6
Sample total/average	136	8.48 (4.52)	0.50–22.27	27.9 (9.18)	13.7–60.1	19.3 (3.03)	11.1–28.0

*Note*: GDP = gross domestic product; LTC = long-term care; n/a = not applicable; PPP = Purchasing Power Parity. Authors’ calculations based on Eurostat (2019) regional data. Statistics calculated from the sample of 136 regions.

**Figure 1.**
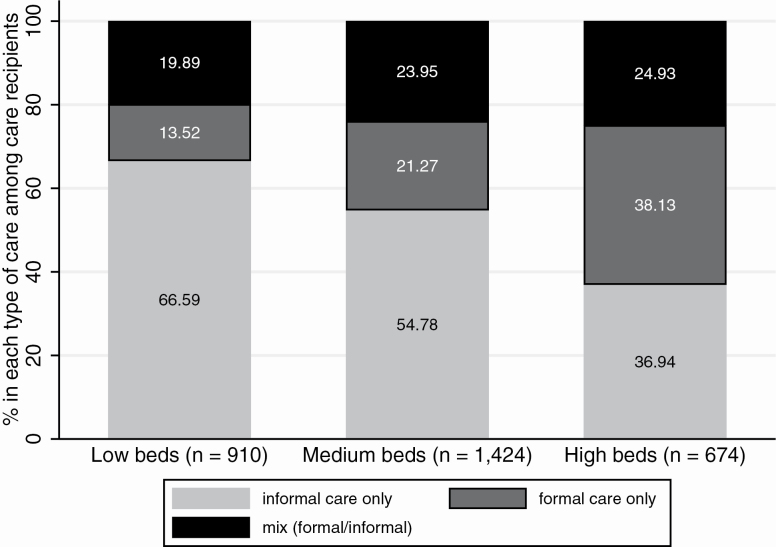
Percentages of care recipients receiving each type of care by LTC beds tertile group. LTC beds tertiles calculated over the sample of regions (*n* = 136). *Note*: LTC = long-term care; The percentages receiving any home-care in each group of regions are: 20.61% (low beds); 20.54% (medium beds); 16.62% (high beds).

In Figure 1, we describe the patterns of care utilization in our data by splitting our sample of respondents into three groups defined by regional-level tertiles of number of LTC beds. We then compare the relative allocation of informal, formal and mixed-care among care recipients in each group of regions. We find that, among care recipients, the percentage of respondents using any formal care (i.e., formal only or mixed) is nearly twice as high in regions with many LTC beds (>11.1/1,000) than in regions with few LTC beds (<7/1,000). Conversely, care recipients in regions with few LTC beds are most likely to rely exclusively on informal care.

When looking jointly at all sources of care (informal and/or formal), we note that the share of respondents receiving any care is lower in regions with high LTC beds (17%) compared to regions with low or medium LTC beds (21%). These descriptive differences may be related to population characteristics (e.g., health and age), summarized for our sample in Supplementary Table 2. In our models, we control for a large set of individual sociodemographic characteristics and for country fixed-effects, which account for other unobserved compositional differences.

### Baseline Model

The results from the baseline random-effects model (M1b) are reported in Supplementary Table 3, and confirm the theoretical predictions (Albertini and Pavolini, 2017; Andersen and Newman, 2005; Broese van Groenou et al., 2006). Poorer individuals are more likely to exclusively use informal care in comparison to those with higher wealth, and individuals with lower incomes are less likely to use only formal home-care than wealthier individuals. These findings are robust to including country fixed-effects and regional covariates (M2b, Supplementary Table 4). In the random-effects model M1b, individuals in regions with more LTC beds are, on average, less likely to rely exclusively on informal care, and more likely to rely on formal-only or mixed home-care, in line with previous literature (Suanet et al., 2012) and descriptive evidence (Figure 1). However, this finding is not robust when accounting for country fixed-effects and regional controls (Supplementary Table 4). In model M2b, there is no significant association between LTC beds and the probability of receiving each type of care.

### Interaction Models

We first estimate models without country fixed-effects, where we interact the indicator for LTC beds with wealth (M1w) and income (M1i), respectively. We then estimate models M2w and M2i, where we additionally include country fixed-effects and regional controls. We separately report coefficients on the interaction effect between LTC beds and wealth (Table 3) or income (Table 4), for each care-type outcome. In order to interpret interaction coefficients in non-linear models, we calculate average marginal effects (AMEs), which indicate the predicted change in the probability of receiving each type of care corresponding to a unit change in the variable of interest (log-wealth or log-income). We compute AMEs at three levels of LTC beds:

**Table 3.** Wealth Inequalities: Average Marginal Effects (AMEs) for Financial Wealth at Specified Levels of Long-term Care (LTC) Beds From Fully Adjusted Models of Home Care Use

	(a) Informal only	(b) Formal only	(c) Mixed
Type of care	AME (95% CI)	AME (95% CI)	AME (95% CI)
M1w (country and region random intercepts)			
Log-wealth			
At beds = 2.55	**−0.002** (**−0.004**, **−0.001**)	0.000 (−0.000, 0.001)	**0.002** (**0.001**, **0.003**)
At beds = 9.10	**−0.002** (**−0.003**, **−0.000**)	0.001 (−0.000, 0.001)	0.000 (−0.001, 0.001)
At beds = 12.43	−0.001 (−0.003, 0.000)	0.001 (−0.001, 0.002)	**−0.001** (**−0.002**, **0.000**)
Individual controls	Yes	Yes	Yes
Regional controls	No	No	No
*n* (individuals)	15,403	15,403	15,403
*n* (regions)	136	136	136
*n* (countries)	12	12	12
M2w (region random intercepts and country fixed-effects)			
Log-wealth			
At beds = 2.55/1,000	**−0.002** (**−0.004**, **−0.001**)	0.000 (−0.001, 0.002)	**0.002** (**0.001**, **0.004**)
At beds = 9.10/1,000	**−0.001** (**−0.002**, **−0.000**)	0.000 (−0.001, 0.001)	0.000 (−0.001, 0.001)
At beds = 12.43/1,000	−0.001 (−0.002, 0.001)	0.000 (−0.001, 0.001)	**−0.001** (**−0.002**, **−0.000**)
Individual controls	Yes	Yes	Yes
Regional controls	Yes	Yes	Yes
*n* (individuals)	14,730	14,730	14,730
*n* (regions)	129	129	129
*n* (countries)	11	11	11

*Note*: All covariates fixed at observed values, and random parameters integrated out. 95% confidence intervals (CIs) in parentheses. AMEs highlighted in bold if 95% CI does not include 0 (*p* < .05). Individual controls: sex, age, activities of daily living, instrumental activities of daily living, mobility, chronic conditions, self-rated health, cognitive function, education, marital status, parent and child coresidence status, home ownership, and access to car. Regional controls: GDP per inhabitant (Purchasing Power Parity-adjusted), percentage of population aged 65+ over total.

**Table 4.** Income Inequalities: Average Marginal Effects (AMEs) for Income at Specified Levels of Long-term Care (LTC) Beds From Fully Adjusted Models of Home Care Use

	(a) Informal only	(b) Formal only	(c) Mixed
Type of care	AME (95% CI)	AME (95% CI)	AME (95% CI)
M1i (country and region random intercepts)			
Log-income			
At beds = 2.55/1,000	−0.006 (−0.016, 0.003)	**0.006 (0.001**, **0.010)**	**0.008 (0.003**, **0.013)**
At beds = 9.10/1,000	**−0.005 (−0.010**, **−0.000)**	**0.006 (0.003**, **0.009)**	**0.003 (0.000**, **0.006)**
At beds = 12.43/1,000	−0.004 (−0.010, 0.002)	**0.005 (0.001**, **0.010)**	−0.001 (−0.005, 0.003)
Individual controls	Yes	Yes	Yes
Regional controls	No	No	No
*n* (individuals)	15,403	15,403	15,403
*n* (regions)	136	136	136
*n* (countries)	12	12	12
M2i (region random intercepts and country fixed-effects)			
Log-income			
At beds = 2.55/1,000	**−0.009 (−0.018**, **−0.000)**	**0.017 (0.004**, **0.031)**	0.005 (−0.004, 0.014)
At beds = 9.10/1,000	−0.005 (−0.012, 0.002)	**0.007 (0.002**, **0.012)**	0.000 (−0.005, 0.004)
At beds = 12.43/1,000	−0.003 (−0.013, 0.007)	0.003 (−0.003, 0.008)	−0.003 (−0.009, 0.003)
Individual controls	Yes	Yes	Yes
Regional controls	Yes	Yes	Yes
*n* (individuals)	14,730	14,730	14,730
*n* (regions)	129	129	129
*n* (countries)	11	11	11

*Note*: All covariates fixed at observed values, and random parameters integrated out. 95% confidence intervals (CIs) in parentheses. AMEs highlighted in bold if 95% CI does not include 0 (*p* < .05). Individual controls: sex, age, activities of daily living, instrumental activities of daily living, mobility, chronic conditions, self-rated health, cognitive function, education, marital status, parent and child coresidence status, home ownership, and access to car. Regional controls: GDP per inhabitant (Purchasing Power Parity-adjusted), percentage of population aged 65+ over total.

- Low: 2.55/1,000 = 20th percentile (e.g., Lower Silesia, Poland);
- Intermediate: 9.10/1,000 = median (e.g., Trento, Italy);
- High: 12.43/1,000 = 80th percentile (e.g., Lower Normandy, France).

In computing AMEs, all other covariates are held at their observed values and random parameters are integrated out.

### Informal Care Only

#### Wealth

Table 3 shows that poorer individuals are more likely than wealthier individuals to rely exclusively on informal care (M1w, panel a). An increase in financial wealth by about 10% corresponds to a decrease in the probability of receiving only informal care by 0.01–0.02 percentage points (p.p.) (see Supplementary Appendix 2). These marginal changes are small when compared to the average probability of receiving only informal care (10.6%). Moreover, such associations are not statistically different across levels of LTC beds (the confidence intervals largely overlap), failing to substantiate H-a in relation to wealth. This result is robust to adding country fixed-effects and regional-level controls to the model (M2w, panel a).

#### Income

For income (Table 4), the results from the random-intercepts model (M1i, panel a) confirm the existence of a pro-poor gradient in informal care use, however with weaker statistical significance. As before, such inequalities do not change with the level of LTC beds, not substantiating H-a. Income gradients in informal care use are more pro-poor in regions with fewer beds when country fixed-effects and regional controls are included (M2i, panel a). However, the confidence intervals for different regions largely overlap. Comparing the estimated AMEs suggests that informal care use is more strongly (negatively) associated with wealth than it is with income, in line with H-d.

### Formal Care Only

#### Wealth

Wealth is not associated with the probability of receiving only formal care, regardless of LTC beds and model specification (Table 3: M1w and M2w, panel b). Thus, we find no evidence for H-b in relation to wealth inequalities.

#### Income

As shown in Table 4, formal home-care use has a significant pro-rich income gradient, with a 10% increase in income corresponding to a 0.05–0.06 p.p. increase in the probability of relying exclusively on formal care, relative to an average probability of formal care use of 4.4%. However, such inequalities do not vary with LTC beds, therefore not substantiating H-b. In the fixed-effects model (M2i, panel b) income gradients in formal care are more strongly pro-rich in regions with fewer beds, but the confidence intervals overlap, not fully substantiating H-b. As hypothesized (H-e), income has a stronger (positive) association with exclusive formal care use than wealth.

### Mixed-Care

#### Wealth

We find a significant interaction between wealth and LTC beds for mixed-care use (Table 3: M1w, panel c). In regions with low numbers of LTC beds, we find pro-rich inequalities, namely a 0.02 p.p. increase in the probability of mixed-care use for a 10% increase in wealth (the average prevalence of mixed-care use is 4.5%); while in regions with high LTC beds, the gradient is pro-poor (0.01 p.p. decrease in care use probability for a 10% increase in wealth). These results are robust to adding country fixed-effects and regional controls (M2w, panel c), and strongly support H-c in relation to wealth inequalities.

#### Income

Results for income are in line with those for wealth in the model with random-effects only, as we find pro-rich income gradients in mixed-care use in regions with low and intermediate LTC beds, while no socioeconomic gradient exists in regions with high LTC beds (Table 4: M1i, panel c). However, when adding country fixed-effects and regional controls, differences in income gradients are no longer significant across levels of LTC beds (M2i, panel c), suggesting that they may be driven by differences in other macro-level factors.

### Sensitivity Analyses

We check the robustness of the estimated standard errors by performing bootstrapping with 100 sample replications. For the resampling, individuals are clustered within regions to preserve the multilevel structure of the data. Supplementary Tables 5 and 6 compare robust and bootstrapped standard errors for the main coefficients of interest, and strongly confirm our main results.

Our analytic sample includes respondents who report difficulties with any of a set of activities including ADLs, IADLs and mobility tasks (see Supplementary Appendix 1). In Supplementary Tables 7–12, we show that our results are robust to alternative definitions of disability, such as (a) at least one IADL or ADL limitation, (b) at least one ADL limitation, and (c) at least two ADL limitations.

Finally, the coefficients on cross-level interactions may be biased as the random-intercept model implicitly assumes that all the variation in wealth or income gradients is explained by LTC beds (Heisig and Schaeffer, 2019). To account for unobserved heterogeneity in the effect of wealth/income across regions, we add random slopes for wealth and income to the models with country fixed-effects and regional controls (Supplementary Table 13). Our main results are confirmed, with pro-rich wealth inequalities in mixed-care in regions with fewer beds, and no wealth inequalities in regions with more beds. The results for income are unchanged when adding a random slope to the model. We additionally find pro-rich wealth gradients in exclusive formal care use that are not detected in previous models (not different across levels of LTC availability, and only significant for regions with intermediate LTC beds).

## Discussion

This study has assessed whether socioeconomic inequalities in the use of informal, formal, and mixed home-care vary across European regions with different levels of de-familization in LTC. Hypotheses H-a and H-b are not supported by the analysis. While poorer individuals seem more likely to rely exclusively on informal care from kin and non-kin, this result is independent of the number of LTC beds by region. Similarly, we find a pro-rich income gradient in formal care use in line with our expectation that income is linked with the ability to purchase formal care. However, this gradient is independent of regional-level indicators of LTC de-familization.

In support of hypothesis H-c, we find that wealth gradients in mixed-care vary by the regional number of LTC beds. In regions with more LTC beds (indicating higher de-familization), wealth gradients in mixed-care are pro-poor, whereas pro-rich gradients are found in regions with fewer LTC beds. While the magnitude of these gradients is small in absolute terms, they are statistically significant and in opposite directions. Importantly, they have been estimated controlling for a wide range of individual and macro-level characteristics, including indicators of health and SES. The same result is found for income, but not confirmed under stricter model specifications. Consistent with hypotheses H-d and H-e, income has a stronger association with formal care, while wealth has a stronger association with informal and mixed-care.

Our study has several limitations. First, since SHARE targets community-dwelling individuals, we are unable to study inequalities in the use of care homes, which is strongly related to de-familization in LTC. Second, our indicator of de-familization in LTC systems does not distinguish between privately paid and publicly provided or subsidized LTC beds. Therefore, we cannot draw conclusions about the implications of public care for inequalities in (in)formal home-care use. Third, as previously noted, our LTC beds indicator does not capture de-familization through formal home-care provision. This might affect our findings to the extent to which the number of LTC beds represent the “flip-side” of availability of formal home-care (i.e., if regions with higher LTC beds have lower levels of formal home-care). However, this is unlikely to be a concern in our setting, for two main reasons. First, at country level, LTC beds are positively correlated with public home-care expenditure in our sample of countries (Supplementary Table 1a and b); in our analyses, we assume the same to be true at the regional level. Second, if a higher number of LTC beds actually indicated lower de-familization across regions, then we would expect to find that the rich are more likely than the poor to use home-care when LTC beds are higher (as formal home-care availability would be lower). In other words, we would find inequalities in mixed-care to be more “pro-rich” in regions with higher LTC beds than in regions with lower LTC beds. However, we find an opposite result, in that inequalities in mixed-care use are more “pro-poor” when LTC beds are higher. This suggests that our estimates of how SES inequalities change when de-familization increases may actually be conservative. Ideally, future comparative research on care-use inequalities would benefit from considering a broader range of LTC system indicators, including home-care expenditure and the availability and amount of cash-for-care benefits. Unfortunately, these data are not currently available at regional level. Moreover, while LTC policies in many European countries are implemented at the NUTS-3 level (European Commission, 2018), comparative data on LTC beds are not available at this level of aggregation.

In terms of data limitations, SHARE is not designed to be representative of regional populations and, for some of the countries under study, some regions are not represented (e.g., Aosta Valley in Italy). Finally, the cross-sectional design of our study does not allow for investigating the implications of policy changes for inequalities in care use, which is a fruitful avenue for future research.

This study advances research that theoretically links inequality in care use to LTC system characteristics, in particular the degree to which alternatives are provided that reduce family responsibilities for care (Saraceno, 2010, 2016). It represents the first study to formally test for interactions between individual SES and contextual features of LTC systems in relation to (in)formal care use. We show that de-familization in LTC relates to socioeconomic gradients in the use of formal and informal care combined (mixed-care). This finding is important at a time when European countries are progressively shifting care responsibilities toward families in order to minimize the rise in LTC costs associated with population ageing (Colombo et al., 2011; European Commission, 2018), raising issues of limited LTC coverage for dependent older people (Brugiavini et al., 2017; Hashiguchi and Llena-Nozal, 2020). Crucially, if mixed-care is a preferred or more beneficial option among older disabled adults (Pinquart and Sörensen, 2002), greater reliance on families for LTC provision may act to widen socioeconomic disparities in health and well-being among older Europeans (Bonsang, 2009).

## Funding

This work was supported by the Economic and Social Research Council (ESRC) (grant number: ES/S01523X/1).

## Conflict of Interest

None declared.

## Supplementary Material

gbaa139_suppl_Supplementary-Material

## Footnotes

^1^ Since Estonia is not divided into NUTS-2 regions, we consider the whole country as a single region. This implies that we cannot distinguish the country-effect from that of LTC beds for Estonia. However, replicating all the analyses excluding Estonia from the sample does not change the results. Therefore, we include it to maximize the sample size.

^2^ We restrict informal care to care received “at least once a week” following Michaud et al. (2010) who define meaningful caregiving as 5 hr per week.

^3^ Only 2013 data are available for France, and only 2011 data for Belgium. We use these figures since equivalent indicators at the national level show no substantial change between these years and 2015 for either country (Eurostat, 2019).
