# Supplementary material for: Socioeconomic Inequalities in Home-Care Use Across Regional Long-term Care Systems in Europe
Source: J Gerontol B Psychol Sci Soc Sci. 2020 Sep 30;76(1):121–32. doi: 10.1093/geronb/gbaa139 (PMC7756692; doi:10.1093/geronb/gbaa139)
Supplement: gbaa139_suppl_Supplementary-Material [file gbaa139_suppl_supplementary-material.docx]

**Socio-economic inequalities in home-care use across regional long-term care systems in Europe**

**SUPPLEMENTARY MATERIAL**

**Supplementary table 1a**. Public expenditure on LTC at home and in institutions as % of GDP and LTC beds (2015)

| **Country** | **LTC expenditure (home care)** | **LTC expenditure (institutions)** | **LTC beds per 1000 inhabitants** |
| --- | --- | --- | --- |
| Austria | 0.7 | 0.8 | 7.77 |
| Belgium | 1.0 | 1.4 | 12.34 |
| Czech Republic | 0.1 | 0.8 | 6.55 |
| Estonia | 0.0 | 0.3 | 8.28 |
| France | 0.3 | 1.5 | 9.68 |
| Germany | 0.8 | 1.0 | 11.37 |
| Italy | 0.1 | 0.5 | 3.93 |
| Poland | 0.3 | 0.1 | 1.84 |
| Spain | 0.2 | 0.6 | 8.13 |
| Sweden | 0.9 | 1.9 | 12.76 |
| Switzerland | 0.3 | 1.9 | 11.68 |

*Note*: only countries in our sample are considered.

Sources: OECD (2020) (<https://www.oecd.org/health/health-systems/health-data.htm>); Eurostat (2020) (<http://appsso.eurostat.ec.europa.eu/nui/submitViewTableAction.do>)

**Supplementary table 1b.** Pairwise correlations among different types of public LTC expenditure and LTC beds

|  | **Home care** | **Institutional care** | **LTC beds** |
| --- | --- | --- | --- |
| **Home care** | 1 |  |  |
| **Institutional care** | 0.53 | 1 |  |
| **LTC beds** | 0.62 | 0.83 | 1 |

Sources: OECD (2020) (<https://www.oecd.org/health/health-systems/health-data.htm>); Eurostat (2020) (<http://appsso.eurostat.ec.europa.eu/nui/submitViewTableAction.do>)

**Supplementary table 2:** Descriptive sample characteristics, overall and by LTC beds tertile group. LTC beds tertiles calculated over the sample of regions (n = 136).

|  |  | **Total** | **By LTC beds tertile group (regions):** | | |
| --- | --- | --- | --- | --- | --- |
|  |  |  | **Low**  **(0.5–7.0)** | **Medium (7.1–11.0)** | **High**  **(11.0–22.3)** |
| **Care receipt** |  |  |  |  |  |
| Informal only | % | 10.6 | 13.7 | 11.3 | 6.1 |
| Formal only | % | 4.5 | 2.8 | 4.4 | 6.4 |
| Mixed care | % | 4.5 | 4.1 | 4.9 | 4.1 |
| **Predisposing factors** |  |  |  |  |  |
| Sex: female | % | 62.5 | 62.8 | 63.1 | 61.1 |
| Age | mean | 75.7 | 75.1 | 76.1 | 75.7 |
| **Need factors** |  |  |  |  |  |
| ADL limitations: none | % | 73.6 | 74.3 | 73.0 | 73.9 |
| 1 limitation | % | 13.2 | 11.2 | 13.0 | 15.6 |
| 2+ limitations | % | 13.2 | 14.5 | 14.0 | 10.5 |
| IADL limitations: none | % | 63.3 | 63.6 | 61.8 | 65.4 |
| 1 limitation | % | 17.0 | 15.4 | 17.3 | 18.5 |
| 2+ limitations | % | 19.7 | 21.0 | 20.9 | 16.2 |
| No. mobility limitations | mean | 3.50 | 3.79 | 3.61 | 3.01 |
| Any chronic conditions: yes | % | 82.2 | 82.5 | 82.7 | 80.9 |
| Poor self-rated health: yes | % | 21.9 | 26.9 | 23.5 | 13.7 |
| Low cognitive function: yes | % | 7.6 | 9.2 | 7.1 | 6.5 |
| **Social resources** |  |  |  |  |  |
| Education: lower secondary | % | 52.6 | 63.2 | 48.9 | 47.3 |
| upper secondary | % | 31.9 | 25.8 | 36.1 | 31.5 |
| tertiary | % | 15.5 | 11.0 | 15.0 | 21.2 |
|  |  |  |  |  |  |
| Marital status: married | % | 60.8 | 63.35 | 58.8 | 61.8 |
| never married | % | 4.3 | 3.5 | 5.0 | 4.1 |
| separated or divorced | % | 7.8 | 6.3 | 8.0 | 9.1 |
| widowed | % | 27.0 | 26.9 | 28.3 | 25.1 |
|  |  |  |  |  |  |
| Parental status: childless | % | 9.6 | 8.2 | 10.5 | 9.4 |
| children outside household | % | 73.1 | 66.1 | 73.5 | 80.1 |
| coresident child/ren | % | 17.4 | 25.8 | 16.0 | 10.5 |
| **Material resources** |  |  |  |  |  |
| Home ownership: yes | % | 70.7 | 74.0 | 71.7 | 65.2 |
| Access to a car: yes | % | 57.2 | 48.4 | 53.2 | 73.6 |
| **Income and wealth** |  |  |  |  |  |
| Income (PPP-adj.) | mean | 47,301 | 31,904 | 64,809 | 132,859 |
| Financial wealth (PPP-adj.) | mean | 162,213 | 55,462 | 115,324 | 358,551 |
|  |  |  |  |  |  |
| **Sample sizes** |  | 15,403 | 4,415 | 6,933 | 4,055 |
| **Sample proportions** |  |  | 28.7 | 45.0 | 26.3 |

**Supplementary table 3.** Exponentiated coefficients (odds ratios) from random-effects model with no interactions (M1b). Odds ratios refer to the odds of receiving each type of care relative to no care.

| **REFERENCE: no care** | **Informal care only** | **Formal care only** | **Mixed care** |
| --- | --- | --- | --- |
|  | Odds ratio (95% CI) | Odds ratio (95% CI) | Odds ratio (95% CI) |
| Sex: female | **0.841 (0.732 ; 0.966)** | **0.804 (0.660 ; 0.979)** | 1.022 (0.830 ; 1.258) |
| Age | **1.028 (1.018 ; 1.039)** | **1.066 (1.052 ; 1.080)** | **1.076 (1.062 ; 1.092)** |
| ADL limitations: none |  |  |  |
| 1 limitation | **4.509 (3.842 ; 5.293)** | **2.575 (2.062 ; 3.215)** | **5.196 (3.884 ; 6.953)** |
| 2+ limitations | **6.701 (5.598 ; 8.022)** | **4.193 (3.273 ; 5.372)** | **14.94 (11.24 ; 19.84)** |
| IADL limitations: none |  |  |  |
| 1 limitation | **2.540 (2.130 ; 3.028)** | **1.837 (1.431 ; 2.359)** | **2.146 (1.489 ; 3.093)** |
| 2+ limitations | **5.139 (4.265 ; 6.192)** | **5.239 (4.076 ; 6.733)** | **9.664 (6.957 ; 13.43)** |
| No. mobility limitations | **1.138 (1.102 ; 1.175)** | **1.067 (1.021 ; 1.115)** | **1.165 (1.111 ; 1.220)** |
| Any chronic conditions: yes | **1.349 (1.089 ; 1.671)** | 1.103 (0.852 ; 1.427) | 1.361 (0.973 ; 1.904) |
| Poor self-rated health: yes | **1.630 (1.408 ; 1.887)** | **1.245 (1.010 ; 1.535)** | **1.542 (1.252 ; 1.900)** |
| Low cognitive function: yes | **1.382 (1.131 ; 1.689)** | 1.260 (0.968 ; 1.639) | **1.311 (1.002 ; 1.716)** |
| Education: lower secondary |  |  |  |
| upper secondary | 1.064 (0.909 ; 1.246) | 1.068 (0.869 ; 1.314) | 1.232 (0.982 ; 1.546) |
| tertiary | 0.891 (0.716 ; 1.109) | 1.064 (0.809 ; 1.400) | 1.158 (0.848 ; 1.582) |
| Marital status: married |  |  |  |
| never married | **0.570 (0.381 ; 0.853)** | 1.205 (0.763 ; 1.905) | 0.876 (0.528 ; 1.454) |
| separated or divorced | **0.479 (0.358 ; 0.640)** | **1.860 (1.354 ; 2.556)** | **0.539 (0.349 ; 0.833)** |
| widowed | **0.565 (0.476 ; 0.670)** | **1.779 (1.429 ; 2.216)** | **0.573 (0.451 ; 0.730)** |
| Parental status: childless |  |  |  |
| children outside household | 1.037 (0.806 ; 1.336) | 0.637 (0.487 ; 0.834) | **0.715 (0.519 ; 0.985)** |
| coresident child/ren | **1.476 (1.116 ; 1.952)** | **0.289 (0.195 ; 0.429)** | 0.851 (0.586 ; 1.234) |
| Home ownership: yes | 1.045 (0.900 ; 1.213) | **0.929 (0.771 ; 1.120)** | 0.827 (0.674 ; 1.015) |
| Access to a car: yes | 0.992 (0.842 ; 1.167) | **0.552 (0.442 ; 0.691)** | 0.882 (0.696 ; 1.118) |
| Log. of income | 0.968 (0.894 ; 1.048) | **1.175 (1.068 ; 1.292)** | 1.101 (0.996 ; 1.216) |
| Log. of financial wealth | **0.978 (0.963 ; 0.994)** | 1.012 (0.990 ; 1.035) | 1.003 (0.980 ; 1.028) |
|  |  |  |  |
| **LTC beds (1000/pop)** | **0.959 (0.927 ; 0.991)** | **1.087 (1.048 ; 1.127)** | **1.037 (0.999 ; 1.076)** |
|  |  |  |  |
| **Sample size** | 15,403 | | |
| $\boldsymbol{\sigma}_{\boldsymbol{u}\boldsymbol{1}}^{\boldsymbol{2}}$**(country-level variance)** | 0.123 (0.062) | | |
| $\boldsymbol{\sigma}_{\boldsymbol{u}\boldsymbol{2}}^{\boldsymbol{2}}$**(region-level variance)** | 0.103 (0.030) | | |

*Note*: 95% Confidence Intervals (CIs) in parentheses. Odds ratios highlighted in bold if 95% CI does not include 0 (p < 0.05).

**Supplementary table 4.** Exponentiated coefficients (odds ratios) from fixed-effects model with no interactions (M2b). Odds ratios refer to the odds of receiving each type of care relative to no care.

| **REFERENCE: no care** | **Informal care only** | **Formal care only** | **Mixed care** |
| --- | --- | --- | --- |
|  | Odds ratio (95% CI) | Odds ratio (95% CI) | Odds ratio (95% CI) |
| Sex: female | **0.848 (0.736 ; 0.977)** | **0.789 (0.642 ; 0.968)** | 1.084 (0.875 ; 1.344) |
| Age | **1.030 (1.020 ; 1.041)** | **1.067 (1.052 ; 1.082)** | **1.072 (1.057 ; 1.088)** |
| ADL limitations: none |  |  |  |
| 1 limitation | **4.647 (3.946 ; 5.473)** | **2.559 (2.033 ; 3.221)** | **5.431 (4.026 ; 7.328)** |
| 2+ limitations | **6.982 (5.819 ; 8.377)** | **3.886 (3.003 ; 5.029)** | **15.61 (11.66 ; 20.91)** |
| IADL limitations: none |  |  |  |
| 1 limitation | **2.552 (2.133 ; 3.052)** | **1.642 (1.263 ; 2.135)** | **2.111 (1.451 ; 3.071)** |
| 2+ limitations | **5.404 (4.472 ; 6.530)** | **4.746 (3.661 ; 6.154)** | **9.430 (6.722 ; 13.23)** |
| No. mobility limitations | **1.133 (1.097 ; 1.170)** | **1.070 (1.022 ; 1.119)** | **1.149 (1.095 ; 1.206)** |
| Any chronic conditions: yes | **1.364 (1.097 ; 1.697)** | **1.061 (0.809 ; 1.392)** | **1.255 (0.887 ; 1.776)** |
| Poor self-rated health: yes | **1.515 (1.305 ; 1.758)** | **1.485 (1.191 ; 1.850)** | **1.606 (1.291 ; 1.998)** |
| Low cognitive function: yes | **1.365 (1.114 ; 1.672)** | **1.324 (1.011 ; 1.735)** | 1.310 (0.994 ; 1.727) |
| Education: lower secondary |  |  |  |
| upper secondary | 1.079 (0.915 ; 1.273) | 1.013 (0.809 ; 1.268) | 1.144 (0.890 ; 1.469) |
| tertiary | 0.944 (0.755 ; 1.181) | 0.979 (0.735 ; 1.303) | 1.178 (0.849 ; 1.636) |
| Marital status: married |  |  |  |
| never married | **0.556 (0.370 ; 0.834)** | 1.103 (0.684 ; 1.781) | 0.759 (0.446 ; 1.292) |
| separated or divorced | **0.478 (0.356 ; 0.642)** | **1.808 (1.297 ; 2.522)** | **0.535 (0.340 ; 0.842)** |
| widowed | **0.544 (0.457 ; 0.647)** | **1.722 (1.370 ; 2.165)** | **0.545 (0.424 ; 0.700)** |
| Parental status: childless |  |  |  |
| children outside household | 0.996 (0.772 ; 1.285) | **0.609 (0.460 ; 0.806)** | **0.684 (0.490 ; 0.956)** |
| coresident child/ren | **1.401 (1.056 ; 1.858)** | **0.276 (0.183 ; 0.418)** | 0.807 (0.547 ; 1.190) |
| Home ownership: yes | 0.966 (0.827 ; 1.128) | 0.993 (0.812 ; 1.215) | 0.942 (0.753 ; 1.178) |
| Access to a car: yes | 1.073 (0.908 ; 1.269) | **0.451 (0.356 ; 0.572)** | **0.775 (0.603 ; 0.997)** |
| Log. of income | 0.947 (0.864 ; 1.037) | **1.172 (1.021 ; 1.346)** | 1.022 (0.884 ; 1.181) |
| Log. of financial wealth | **0.980 (0.965 ; 0.996)** | 1.004 (0.981 ; 1.028) | 0.998 (0.974 ; 1.024) |
|  |  |  |  |
| LTC beds (1000/pop) | 0.992 (0.951 ; 1.034) | 0.976 (0.926 ; 1.029) | 0.975 (0.924 ; 1.028) |
| GDP per capita (1000s) | 0.999 (0.986 ; 1.012) | 0.986 (0.972 ; 1.001) | 1.005 (0.989 ; 1.022) |
| % pop 65+ | 1.025 (0.971 ; 1.083) | 1.016 (0.950 ; 1.086) | **1.086 (1.012 ; 1.165)** |
|  |  |  |  |
| **Sample size** | 14,730 | | |
| $\boldsymbol{\sigma}_{\boldsymbol{u}}^{\boldsymbol{2}}$**(region-level variance)** | 0.073 (0.025) | | |

*Note*: 95% Confidence Intervals (CIs) in parentheses. Odds ratios highlighted in bold if 95% CI does not include 0 (p < 0.05).

**Supplementary table 5.** Comparison between robust and bootstrapped standard errors for the models with wealth interactions. Bootstrap sample N = 100.

| **Variable** | **Coefficient** | **Robust S.E.** | **Bootstrapped S.E.** |
| --- | --- | --- | --- |
| **Random-effects model (M1w), n = 15,403** | | | |
| **1. Informal care only** | | | |
| Log wealth | - 0.013 | 0.016 | 0.015 |
| LTC beds | - 0.033 | 0.023 | 0.021 |
| Log wealth*LTC beds | - 0.001 | 0.002 | 0.002 |
| **2. Formal care only** | | | |
| Log wealth | 0.027 | 0.026 | 0.033 |
| LTC beds | 0.099 | 0.029 *** | 0.039 ** |
| Log wealth*LTC beds | - 0.002 | 0.003 | 0.003 |
| **3. Mixed-care** | | | |
| Log wealth | 0.081 | 0.025 *** | 0.026 ** |
| LTC beds | 0.110 | 0.028 *** | 0.031 *** |
| Log wealth*LTC beds | - 0.009 | 0.003 *** | 0.003 *** |
| **Fixed-effects model (M2w), n = 14,730** | | | |
| **1. Informal care only** | | | |
| Log wealth | - 0.022 | 0.016 | 0.016 |
| LTC beds | - 0.011 | 0.026 | 0.032 |
| Log wealth*LTC beds | 0.001 | 0.002 | 0.002 |
| **2. Formal care only** | | | |
| Log wealth | 0.022 | 0.028 | 0.022 |
| LTC beds | - 0.007 | 0.036 | 0.043 |
| Log wealth*LTC beds | - 0.002 | 0.003 | 0.004 |
| **3. Mixed-care** | | | |
| Log wealth | 0.074 | 0.027 ** | 0.026 ** |
| LTC beds | 0.041 | 0.034 | 0.046 |
| Log wealth*LTC beds | - 0.009 | 0.003 *** | 0.003 *** |

*** p <0.001, ** p < 0.01, * p < 0.05, ~ p < 0.10

**Individual controls**: sex, age, ADL, IADL, mobility, chronic conditions, self-rated health, cognitive function, education, marital status, parent and child coresidence status, home ownership, access to car. **Regional controls**: GDP per inhabitant (PPP-adjusted), percentage of population aged 65+ over total.

**Supplementary table 6.** Comparison between robust and bootstrapped standard errors for the models with income interactions. Bootstrap sample N = 100.

| **Variable** | **Coefficient** | **Robust S.E.** | **Bootstrapped S.E.** |
| --- | --- | --- | --- |
| **Random-effects model (M1i), n = 15,403** | | | |
| **1. Informal care only** | | | |
| Log income | 0.008 | 0.086 | 0.089 |
| LTC beds | 0.005 | 0.098 | 0.110 |
| Log income*LTC beds | - 0.005 | 0.009 | 0.011 |
| **2. Formal care only** | | | |
| Log income | 0.316 | 0.123 ** | 0.231 |
| LTC beds | 0.261 | 0.127 * | 0.255 |
| Log income*LTC beds | - 0.017 | 0.012 | 0.023 |
| **3. Mixed-care** | | | |
| Log income | 0.381 | 0.125 ** | 0.015 ** |
| LTC beds | 0.365 | 0.135 ** | 0.146 * |
| Log income*LTC beds | - 0.032 | 0.130 * | 0.014 * |
| **Fixed-effects model (M2i), n = 14,730** | | | |
| **1. Informal care only** | | | |
| Log income | - 0.043 | 0.090 | 0.087 |
| LTC beds | 0.002 | 0.110 | 0.104 |
| Log income*LTC beds | - 0.001 | 0.011 | 0.010 |
| **2. Formal care only** | | | |
| Log income | 0.501 | 0.171 ** | 0.259 * |
| LTC beds | 0.356 | 0.168 * | 0.280 |
| Log income*LTC beds | - 0.037 | 0.016 * | 0.026 |
| **3. Mixed-care** | | | |
| Log income | 0.268 | 0.128 * | 0.165 ~ |
| LTC beds | 0.273 | 0.170 | 0.170 |
| Log income*LTC beds | - 0.030 | 0.017 ~ | 0.017 ~ |

*** p <0.001, ** p < 0.01, * p < 0.05, ~ p < 0.10

**Individual controls**: sex, age, ADL, IADL, mobility, chronic conditions, self-rated health, cognitive function, education, marital status, parent and child coresidence status, home ownership, access to car. **Regional controls**: GDP per inhabitant (PPP-adjusted), percentage of population aged 65+ over total.

**Supplementary table 7.** IADL/ADL sample: Average marginal effects (AMEs) for financial wealth at specified levels of LTC beds from fully-adjusted models of care receipt. All covariates fixed at observed values, and random parameters integrated out.

| **Type of care** | **Informal only** | **Formal only** | **Mixed** |
| --- | --- | --- | --- |
|  | AME (95% CI) | AME (95% CI) | AME (95% CI) |
| **M1w (country and region random intercepts)** | | | |
| Log wealth |  |  |  |
| at beds = 2.55 | - 0.004 (- 0.007 ; 0.000) | 0.001 (- 0.000 ; 0.003) | **0.004 ( 0.002 ; 0.007)** |
| at beds = 9.10 | - 0.003 (- 0.006 ; - 0.001) | 0.001 (- 0.000 ; 0.003) | 0.000 (- 0.001 ; 0.002) |
| at beds = 12.43 | - 0.002 (- 0.006 ; 0.000) | 0.001 (- 0.001 ; 0.004) | - 0.002 (- 0.004 ; 0.000) |
| Individual controls | Yes | Yes | Yes |
| Regional controls | No | No | No |
|  |  |  |  |
| n (individuals) | 6,723 | 6,723 | 6,723 |
| n (regions) | 136 | 136 | 136 |
| n (countries) | 12 | 12 | 12 |
| **M2w (region random intercepts and country fixed-effects)** | | | |
| Log wealth |  |  |  |
| at beds = 2.55/1000 | **- 0.004 (- 0.007 ; - 0.000)** | 0.001 (- 0.003 ; 0.004) | **0.005 ( 0.001 ; 0.008)** |
| at beds = 9.10/1000 | - 0.002 (- 0.005 ; 0.001) | 0.000 (- 0.001 ; 0.002) | 0.000 (- 0.002 ; 0.002) |
| at beds = 12.43/1000 | - 0.001 (- 0.005 ; 0.003) | - 0.000 (- 0.002 ; 0.002) | - 0.002 (- 0.004 ; 0.000) |
| Individual controls | Yes | Yes | Yes |
| Regional controls | Yes | Yes | Yes |
|  |  |  |  |
| n (individuals) | 6,496 | 6,496 | 6,496 |
| n (regions) | 129 | 129 | 129 |
| n (countries) | 11 | 11 | 11 |

*Note*: 95% Confidence Intervals (CIs) in parentheses. AMEs highlighted in bold if 95% CI does not include 0 (p < 0.05). **Individual controls**: sex, age, ADL, IADL, mobility, chronic conditions, self-rated health, cognitive function, education, marital status, parent and child coresidence status, home ownership, access to car. **Regional controls**: GDP per inhabitant (PPP-adjusted), percentage of population aged 65+ over total.

**Supplementary table 8.** IADL/ADL sample: Average marginal effects (AMEs) for income at specified levels of LTC beds from fully-adjusted models of care receipt. All covariates fixed at observed values, and random parameters integrated out.

| **Type of care** | **Informal only** | **Formal only** | **Mixed** |  |
| --- | --- | --- | --- | --- |
|  | AME (95% CI) | AME (95% CI) | AME (95% CI) |  |
| **M1i (country and region random intercepts)** | | | |  |
| Log income |  |  |  |  |
| at beds = 2.55/1000 | 0.008 (- 0.013 ; 0.029) | **0.009 ( 0.001 ; 0.017)** | **0.022 ( 0.011 ; 0.034)** |  |
| at beds = 9.10/1000 | **- 0.012 (- 0.022 ; - 0.002)** | **0.012 ( 0.006 ; 0.018)** | **0.008 ( 0.002 ; 0.014)** |  |
| at beds = 12.43/1000 | **- 0.018 (- 0.031 ; - 0.004)** | **0.012 ( 0.003 ; 0.021)** | - 0.002 (- 0.012 ; 0.007) |  |
| Individual controls | Yes | Yes | Yes |  |
| Regional controls | No | No | No |  |
|  |  |  |  |  |
| n (individuals) | 6,723 | 6,723 | 6,723 |  |
| n (regions) | 136 | 136 | 136 |  |
| n (countries) | 12 | 12 | 12 |  |
| **M2i (region random intercepts and country fixed-effects)** | | | |  |
| Log income |  |  |  |  |
| at beds = 2.55/1000 | - 0.007 (- 0.027 ; 0.013) | **0.039 ( 0.013 ; 0.066)** | 0.016 (- 0.004 ; 0.036) |  |
| at beds = 9.10/1000 | - 0.012 (- 0.027 ; 0.004) | **0.015 ( 0.005 ; 0.026)** | 0.001 (- 0.009 ; 0.012) |  |
| at beds = 12.43/1000 | - 0.015 (- 0.036 ; 0.007) | 0.005 (- 0.006 ; 0.016) | - 0.006 (- 0.019 ; 0.008) |  |
| Individual controls | Yes | Yes | Yes |  |
| Regional controls | No | No | No |  |
|  |  |  |  |  |
| n (individuals) | 6,496 | 6,496 | 6,496 |  |
| n (regions) | 129 | 129 | 129 |  |
| n (countries) | 11 | 11 | 11 |  |

*Note*: 95% Confidence Intervals (CIs) in parentheses. AMEs highlighted in bold if 95% CI does not include 0 (p < 0.05). **Individual controls**: sex, age, ADL, IADL, mobility, chronic conditions, self-rated health, cognitive function, education, marital status, parent and child coresidence status, home ownership, access to car. **Regional controls**: GDP per inhabitant (PPP-adjusted), percentage of population aged 65+ over total.

**Supplementary table 9.** ADL sample: Average marginal effects (AMEs) for financial wealth at specified levels of LTC beds from fully-adjusted models of care receipt. All covariates fixed at observed values, and random parameters integrated out.

| **Type of care** | **Informal only** | **Formal only** | **Mixed** |
| --- | --- | --- | --- |
|  | AME (95% CI) | AME (95% CI) | AME (95% CI) |
| **M1w (country and region random intercepts)** | | | |
| Log wealth |  |  |  |
| at beds = 2.55 | - 0.003 (- 0.009 ; 0.002) | 0.003 (- 0.000 ; 0.005) | **0.007 ( 0.003 ; 0.011)** |
| at beds = 9.10 | **- 0.004 (- 0.007 ; - 0.001)** | **0.002 ( 0.000 ; 0.005)** | 0.001 (- 0.001 ; 0.004) |
| at beds = 12.43 | - 0.003 (- 0.008 ; 0.001) | 0.002 (- 0.001 ; 0.005) | - 0.003 (- 0.006 ; 0.001) |
| Individual controls | Yes | Yes | Yes |
| Regional controls | No | No | No |
|  |  |  |  |
| n (individuals) | 4,069 | 4,069 | 4,069 |
| n (regions) | 136 | 136 | 136 |
| n (countries) | 12 | 12 | 12 |
| **M2w (region random intercepts and country fixed-effects)** | | | |
| Log wealth |  |  |  |
| at beds = 2.55/1000 | - 0.004 (- 0.009 ; 0.001) | 0.002 (- 0.003 ; 0.007) | **0.008 ( 0.003 ; 0.013)** |
| at beds = 9.10/1000 | - 0.001 (- 0.005 ; 0.002) | 0.001 (- 0.001 ; 0.003) | 0.001 (- 0.002 ; 0.003) |
| at beds = 12.43/1000 | - 0.000 (- 0.005 ; 0.005) | 0.000 (- 0.002 ; 0.003) | - 0.003 (- 0.006 ; 0.001) |
| Individual controls | Yes | Yes | Yes |
| Regional controls | Yes | Yes | Yes |
|  |  |  |  |
| n (individuals) | 3,938 | 3,938 | 3,938 |
| n (regions) | 129 | 129 | 129 |
| n (countries) | 11 | 11 | 11 |

*Note*: 95% Confidence Intervals (CIs) in parentheses. AMEs highlighted in bold if 95% CI does not include 0 (p < 0.05). **Individual controls**: sex, age, ADL, IADL, mobility, chronic conditions, self-rated health, cognitive function, education, marital status, parent and child coresidence status, home ownership, access to car. **Regional controls**: GDP per inhabitant (PPP-adjusted), percentage of population aged 65+ over total.

**Supplementary table 10.** ADL sample: Average marginal effects (AMEs) for income at specified levels of LTC beds from fully-adjusted models of care receipt. All covariates fixed at observed values, and random parameters integrated out.

| **Type of care** | **Informal only** | **Formal only** | **Mixed** |
| --- | --- | --- | --- |
|  | AME (95% CI) | AME (95% CI) | AME (95% CI) |
| **M1i (country and region random intercepts)** | | | |
| Log income |  |  |  |
| at beds = 2.55/1000 | 0.008 (- 0.019 ; 0.034) | 0.010 (- 0.001 ; 0.021) | **0.031 ( 0.013 ; 0.048)** |
| at beds = 9.10/1000 | **- 0.018 (- 0.032 ; - 0.004)** | **0.014 ( 0.006 ; 0.022)** | 0.008 (- 0.002 ; 0.017) |
| at beds = 12.43/1000 | **- 0.025 (- 0.042 ; - 0.007)** | **0.016 ( 0.005 ; 0.028)** | - 0.010 (- 0.024 ; 0.005) |
| Individual controls | Yes | Yes | Yes |
| Regional controls | No | No | No |
|  |  |  |  |
| n (individuals) | 4,069 | 4,069 | 4,069 |
| n (regions) | 136 | 136 | 136 |
| n (countries) | 12 | 12 | 12 |
| **M2i (region random intercepts and country fixed-effects)** | | | |
| Log income |  |  |  |
| at beds = 2.55/1000 | - 0.017 (- 0.042 ; 0.009) | **0.053 ( 0.019 ; 0.087)** | 0.026 (- 0.004 ; 0.054) |
| at beds = 9.10/1000 | - 0.016 (- 0.038 ; 0.004) | **0.023 ( 0.008 ; 0.037)** | - 0.000 (- 0.017 ; 0.016) |
| at beds = 12.43/1000 | - 0.018 (- 0.048 ; 0.012) | 0.010 (- 0.005 ; 0.025) | - 0.014 (- 0.034 ; 0.007) |
| Individual controls | Yes | Yes | Yes |
| Regional controls | Yes | Yes | Yes |
|  |  |  |  |
| n (individuals) | 3,938 | 3,938 | 3,938 |
| n (regions) | 129 | 129 | 129 |
| n (countries) | 11 | 11 | 11 |

*Note*: 95% Confidence Intervals (CIs) in parentheses. AMEs highlighted in bold if 95% CI does not include 0 (p < 0.05). **Individual controls**: sex, age, ADL, IADL, mobility, chronic conditions, self-rated health, cognitive function, education, marital status, parent and child coresidence status, home ownership, access to car. **Regional controls**: GDP per inhabitant (PPP-adjusted), percentage of population aged 65+ over total.

**Supplementary table 11.** 2+ ADL sample: Average marginal effects (AMEs) for financial wealth at specified levels of LTC beds from fully-adjusted models of care receipt. All covariates fixed at observed values, and random parameters integrated out.

| **Type of care** | **Informal only** | **Formal only** | **Mixed** |
| --- | --- | --- | --- |
|  | AME (95% CI) | AME (95% CI) | AME (95% CI) |
| **M1w (country and region random intercepts)** | | | |
| Log wealth |  |  |  |
| at beds = 2.55 | - 0.005 (- 0.012 ; 0.002) | 0.003 (- 0.001 ; 0.007) | **0.009 ( 0.003 ; 0.016)** |
| at beds = 9.10 | **- 0.009 (- 0.014 ; - 0.004)** | **0.004 ( 0.001 ; 0.008)** | 0.003 (- 0.002 ; 0.008) |
| at beds = 12.43 | **- 0.009 (- 0.016 ; - 0.002)** | 0.005 (- 0.000 ; 0.011) | - 0.002 (- 0.008 ; 0.005) |
| Individual controls | Yes | Yes | Yes |
| Regional controls | No | No | No |
|  |  |  |  |
| n (individuals) | 2,038 | 2,038 | 2,038 |
| n (regions) | 136 | 136 | 136 |
| n (countries) | 12 | 12 | 12 |
| **M2w (region random intercepts and country fixed-effects)** | | | |
| Log wealth |  |  |  |
| at beds = 2.55/1000 | - 0.005 (- 0.012 ; 0.002) | 0.002 (- 0.005 ; 0.009) | **0.011 ( 0.003 ; 0.018)** |
| at beds = 9.10/1000 | - 0.004 (- 0.009 ; 0.001) | 0.001 (- 0.002 ; 0.005) | 0.002 (- 0.003 ; 0.006) |
| at beds = 12.43/1000 | - 0.003 (- 0.010 ; 0.004) | 0.001 (- 0.003 ; 0.005) | - 0.002 (- 0.008 ; 0.004) |
| Individual controls | Yes | Yes | Yes |
| Regional controls | Yes | Yes | Yes |
|  |  |  |  |
| n (individuals) | 2,001 | 2,001 | 2,001 |
| n (regions) | 129 | 129 | 129 |
| n (countries) | 11 | 11 | 11 |

*Note*: 95% Confidence Intervals (CIs) in parentheses. AMEs highlighted in bold if 95% CI does not include 0 (p < 0.05). **Individual controls**: sex, age, ADL, IADL, mobility, chronic conditions, self-rated health, cognitive function, education, marital status, parent and child coresidence status, home ownership, access to car. **Regional controls**: GDP per inhabitant (PPP-adjusted), percentage of population aged 65+ over total.

**Supplementary table 12.** 2+ ADL sample: Average marginal effects (AMEs) for income at specified levels of LTC beds from fully-adjusted models of care receipt. All covariates fixed at observed values, and random parameters integrated out.

| **Type of care** | **Informal only** | **Formal only** | **Mixed** |
| --- | --- | --- | --- |
|  | AME (95% CI) | AME (95% CI) | AME (95% CI) |
| **M1i (country and region random intercepts)** | | | |
| Log income |  |  |  |
| at beds = 2.55/1000 | - 0.002 (- 0.039 ; 0.034) | 0.008 (- 0.007 ; 0.024) | **0.036 ( 0.009 ; 0.063)** |
| at beds = 9.10/1000 | **- 0.030 (- 0.050 ; - 0.011)** | **0.020 ( 0.008 ; 0.032)** | 0.011 (- 0.006 ; 0.027) |
| at beds = 12.43/1000 | **- 0.037 (- 0.064 ; - 0.009)** | **0.029 ( 0.011 ; 0.047)** | - 0.010 (- 0.035 ; 0.016) |
| Individual controls | Yes | Yes | Yes |
| Regional controls | No | No | No |
|  |  |  |  |
| n (individuals) | 2,038 | 2,038 | 2,038 |
| n (regions) | 136 | 136 | 136 |
| n (countries) | 12 | 12 | 12 |
| **M2i (region random intercepts and country fixed-effects)** | | | |
| Log income |  |  |  |
| at beds = 2.55/1000 | **- 0.049 (- 0.086 ; - 0.012)** | **0.083 ( 0.038 ; 0.128)** | 0.021 (- 0.022 ; 0.063) |
| at beds = 9.10/1000 | **- 0.039 (- 0.071 ; - 0.007)** | **0.041 ( 0.018 ; 0.063)** | - 0.002 (- 0.029 ; 0.026) |
| at beds = 12.43/1000 | - 0.033 (- 0.078 ; 0.014) | 0.021 (- 0.004 ; 0.046) | - 0.015 (- 0.051 ; 0.022) |
| Individual controls | Yes | Yes | Yes |
| Regional controls | Yes | Yes | Yes |
|  |  |  |  |
| n (individuals) | 2,001 | 2,001 | 2,001 |
| n (regions) | 129 | 129 | 129 |
| n (countries) | 11 | 11 | 11 |

*Note*: 95% Confidence Intervals (CIs) in parentheses. AMEs highlighted in bold if 95% CI does not include 0 (p < 0.05). **Individual controls**: sex, age, ADL, IADL, mobility, chronic conditions, self-rated health, cognitive function, education, marital status, parent and child coresidence status, home ownership, access to car. **Regional controls**: GDP per inhabitant (PPP-adjusted), percentage of population aged 65+ over total.

**Supplementary table 13.** Random slopes model: Average marginal effects (AMEs) for financial wealth and income at specified levels of LTC beds from fully-adjusted models of care receipt. All covariates fixed at observed values, and random parameters integrated out.

| **Type of care** | **Informal only** | **Formal only** | **Mixed** |
| --- | --- | --- | --- |
|  | AME (95% CI) | AME (95% CI) | AME (95% CI) |
| **Wealth (region random intercepts + random slopes for wealth, and country fixed-effects)** | | | |
| Log wealth |  |  |  |
| at beds = 2.55/1000 | - 0.002 (- 0.005 ; 0.002) | 0.002 (- 0.002 ; 0.005**)** | **0.003 ( 0.000 ; 0.006)** |
| at beds = 9.10/1000 | 0.001 (- 0.002 ; 0.003) | **0.002 ( 0.000 ; 0.003)** | 0.000 (- 0.001 ; 0.001) |
| at beds = 12.43/1000 | 0.002 (- 0.001 ; 0.005) | 0.002 (- 0.001 ; 0.004) | - 0.001 (- 0.002 ; 0.001) |
| Individual controls | Yes | Yes | Yes |
| Regional controls | Yes | Yes | Yes |
|  |  |  |  |
| n (individuals) | 14,730 | 14,730 | 14,730 |
| n (regions) | 129 | 129 | 129 |
| n (countries) | 11 | 11 | 11 |
| **Income (region random intercepts + random slopes for income, and country fixed-effects)** | | | |
| Log income |  |  |  |
| at beds = 2.55/1000 | **- 0.017 (- 0.034 ; - 0.000)** | **0.028 ( 0.008 ; 0.048)** | 0.005 (- 0.006 ; 0.015) |
| at beds = 9.10/1000 | - 0.005 (- 0.015 ; 0.005) | **0.015 ( 0.006 ; 0.024)** | 0.000 (- 0.006 ; 0.006) |
| at beds = 12.43/1000 | 0.002 (- 0.012 ; 0.016) | 0.008 (- 0.002 ; 0.018) | - 0.003 (- 0.011 ; 0.005) |
| Individual controls | Yes | Yes | Yes |
| Regional controls | Yes | Yes | Yes |
|  |  |  |  |
| n (individuals) | 14,730 | 14,730 | 14,730 |
| n (regions) | 129 | 129 | 129 |
| n (countries) | 11 | 11 | 11 |

*Note*: 95% Confidence Intervals (CIs) in parentheses. AMEs highlighted in bold if 95% CI does not include 0 (p < 0.05). **Individual controls**: sex, age, ADL, IADL, mobility, chronic conditions, self-rated health, cognitive function, education, marital status, parent and child coresidence status, home ownership, access to car. **Regional controls**: GDP per inhabitant (PPP-adjusted), percentage of population aged 65+ over total.

**Appendix 1: List of activities defining disability in the SHARE sample**

| Activities of Daily Living (ADL)  (total = 6) | Dressing, including putting on shoes and socks  Walking across a room  Bathing or showering  Eating, such as cutting up food  Getting in or out of bed  Using the toilet, including getting up or down |
| --- | --- |
| Instrumental Activities of Daily Living (IADL)  (total = 7) | Using a map to figure out how to get around  Preparing a hot meal  Shopping for groceries  Making telephone calls  Taking medications  Doing work around the house or garden  Managing money, such as bills and expenses |
| Mobility items  (total = 10) | Walking 100 metres  Sitting for about two hours  Getting up from a chair after sitting for long periods  Climbing several flights of stairs without resting  Climbing one flight of stairs without resting  Stooping, kneeling, or crouching  Reaching or extending arms above shoulder level  Pulling or pushing large objects  Lifting or carrying weights over 10 pounds/ 5kg  Picking up a small coin from a table |

**Appendix 2: A note on the interpretation of coefficients on variables transformed using the inverse hyperbolic sine function**

Below is an example of how to interpret the coefficients on a variable that has been transformed using the inverse hyperbolic sine (arcsinh) function. The arcsinh function approximates the natural logarithm, but it is defined for values of a variable that are zero or negative (while the logarithm is not).

Suppose that we transform wealth into log-wealth by taking the natural logarithm. We have log-wealth, log(W), as regressor, and probability of informal care (IC) as the outcome. Say that our estimated marginal effect is 0.002.

This means that $\frac{\partial IC}{\partial\ln\left( W \right)}=0.002$, so an increase in one unit in log(W) corresponds to an increase in 0.002 percentage points in the probability of receiving informal care.

This unitary increase in the log of wealth can be written as $\ln\left( W_{2} \right)-\ln\left( W_{1} \right)=\ln\left( \frac{W_{2}}{W_{1}} \right)=1$ .

Of course, we do not know what $W_{2}$ and $W_{1}$ are, but we know that their log values differ by 1. By exponentiating both sides of the equation, we obtain $\frac{W_{2}}{W_{1}}=e$.

Since $e=2.718,$ $W_{2}= W_{1}* 2.718$ .

Thus, the increase in log(W) by one unit corresponds to an increase in (average) wealth by 172%. I.e. an increase in wealth by 172% corresponds to an increase in probability of IC by 0.002, equivalent to 0.2 percentage points.

In our paper, we are interested in the coefficient for a small increase in wealth (say, 10% increase) rather than a very large increase such as 172%, as the latter may lead to inaccurate predictions for the dependent variable. In order to interpret the marginal effect for a 10% change in wealth, we multiply the marginal effect that we have obtained by 0.1. Note that this is an approximation: the coefficient should be multiplied by log(1.1), which is roughly 0.1. Therefore:

0.002 * 0.1 = 0.0002

A 10% increase in wealth is associated with a 0.0002 increase in IC, which is a 0.02 percentage points increase in the probability of receiving informal care.

**Note that this interpretation also holds when wealth is transformed using the inverse hyperbolic sine function.**

The inverse hyperbolic sine transformation relates to log in the following way:

$arcsinh \left( W \right)\cong\ln\left( 2W \right)$ if W is not too small

Our marginal effect 0.002 represents the change in the probability of informal care when *arcsinh(W)* increases by 1 unit.

Following the same steps as before,

$$arcsinh\left( W_{2} \right)-arcsinh\left( W_{1} \right)\cong\ln\left( 2W_{2} \right)-\ln\left( 2W_{1} \right)=\ln\left( \frac{2W_{2}}{2W_{1}} \right)=\ln\left( \frac{W_{2}}{W_{1}} \right)=1$$

Thus, as before, $\frac{W_{2}}{W_{1}}=e$. So the interpretation of our marginal coefficient is exactly the same as if we had a log transformation. We can follow the same reasoning as before, and multiply the coefficient by 0.1, to get the marginal increase in probability for a (roughly) 10% increase in wealth.
